# Do, don’t and don’t know: guidelines for medical education with a difference

**DOI:** 10.1007/s40037-015-0230-8

**Published:** 2015-10-29

**Authors:** Robert K. McKinley, Fedde Scheele

**Affiliations:** 1 Keele University School of Medicine, ST5 5BG, Keele, Staffordshire UK; 2 VU University, Amsterdam, The Netherlands

As clinician educators, we are busy people. We balance the demands of clinical service, administration, management, education and training. We aim to both meet the expectations of and to achieve the best possible outcomes for our patients, service and learners. Unfortunately, we suspect that many of us find that our educational practice comes last. We have to fit teaching and training around our clinical and administration and management responsibilities. And when it comes to our continuing professional development our learning needs as clinicians must come first.

But we are clinical educators not because we have to be, it is because we want to be. We read the abstracts of papers which offer tantalizing insights into ways in which we could improve our teaching and training. But can we trust the abstract? We set the paper aside on the ever growing pile of papers we must get round to reading one day. And never do.

Of course we have guidelines and systematic reviews to which we could turn. But they are long, heavy in academic detail and unfortunately often heavy to read. And they too get set aside on the pile of papers we must get round to reading one day. And never do.

In this issue we publish the first of a series of ‘Guidelines for medical education’, which we hope will be different. Our aim is that they will succinctly summarize what you should *do* because there is evidence it works, what you should *not do* because there is evidence that it does not work or indeed is likely to cause ‘educational harm’ and things which, although they offer potential, we *do not know* whether they work or not: the ‘Do’s, Don’ts and Don't Knows’ (or ‘D3) of medical education. Initially, we will focus on topics of interest to clinician educators. Each will be written by a small group of authors, authorities in the subject, most of whom are also clinicians. Each D3 will have a short tabulated summary of the Do’s, Don’ts and Don’t Knows’, an indication of the strength of evidence which underpins them and be backed up by an authoritative summary of the underpinning evidence. Our aim is that they will be both accessible to busy clinicians and make a contribution to scholarship: by identifying important ‘Don't Knows’, we hope to stimulate research.

This is an experimental format which is not yet cast in stone—and may never be. Some guidelines may be underpinned by new systematic reviews while some, because of the wealth of existing reviews, will not be. Our methods of indicating the strength of evidence may evolve. As editors we will learn how to support our authors in producing them. The first D3 addresses giving feedback to learners on their performance [1]. We would like to thank Janet Lefroy, Chris Watling, Pim Teunissen and Paul Brand for authoring it and for their patience in working with us to address a target which was probably inadequately drawn initially, and expectations which evolved as we reviewed successive versions. We would also like to thank the authors for developing our ideas, in particular their method of describing the strength of evidence, which is better than what we suggested. We have co-written the instructions for authors with Janet Lefroy to combine her experience as the lead author of the prototype D3 with our expectations as editors.

We aim to publish two each year and we hope you find each D3 accessible, useful and a stimulus for both your educational practice and research. We would value your feedback on the format and the content as we work to make them as useful as possible to you, our readers and colleagues.
